# Exploring Speckle Change Genes of *Rhynchophorus ferrugineus* (Coleoptera: Curculionidae) Based on Genome-Wide Association Studies (GWASs)

**DOI:** 10.3390/biology15070555

**Published:** 2026-03-31

**Authors:** Long Liu, Xin Chen, Cheng Lin, Hua Yang, Qiong Huang, Chunlin Yang, Shujiang Li

**Affiliations:** 1 College of Forestry, Sichuan Agricultural University, Chengdu 611130, China; liulong@sicau.edu.cn (L.L.); sauchenxin@163.com (X.C.);; 2 National Forestry and Grassland Administration Key Laboratory of Forest Resources Conservation and Ecological Safety on the Upper Reaches of the Yangtze River & Forestry Ecological Engineering in the Upper Reaches of the Yangtze River Key Laboratory of Sichuan Province, Chengdu 611130, China

**Keywords:** *Rhynchophorus ferrugineus*, speckle traits, quantitative trait mixed analysis model, whole genome resequencing, GWAS

## Abstract

*Rhynchophorus ferrugineus* is a destructive trunk-boring pest of palm plants and exhibits marked polymorphism in adult speckle patterns. This study investigated the genetic basis and molecular mechanisms underlying speckle variation in *R. ferrugineus* with the aim of identifying key regulatory genes. Integrated genetic analyses and genome-wide association approaches were used to characterize the inheritance and genomic architecture of speckle traits. Pronotum speckle variation was identified as a sex-independent quantitative trait controlled by major loci with additive and dominance effects. Multiple loci and significant quantitative trait nucleotides associated with speckle variation were identified through linkage mapping and association analyses. By integrating genetic mapping results with bioinformatics annotation, three candidate genes, *RferTRXR1*, *RferGH1*, and *RferLAC2*, were identified. Quantitative real-time PCR analysis showed that *RferTRXR1* and *RferGH1* were more highly expressed in individuals with higher speckle numbers. These findings highlight *RferTRXR1* and *RferGH1* as potential regulators of speckle pattern variation in *R. ferrugineus* and provide new insights into the molecular mechanisms underlying phenotypic polymorphism in an important palm pest.

## Simple Summary

The red palm weevil is one of the most damaging pests of palm trees worldwide. In addition to its severe agricultural impact, this insect shows striking variation in body speckles, a visible trait whose genetic basis has been unclear. To uncover how these patterns are controlled, we combined genetic crosses, genome mapping, and population-level association studies. We found that variation in speckle number is influenced by multiple genetic factors, and we identified several key candidate genes linked to melanin production. Two of these genes were more active in insects with many speckles, suggesting they help drive darker pigmentation. By revealing the genetic underpinnings of speckle variation, our work not only provides insights into how insect color traits evolve but also offers new genetic markers that may be useful for monitoring and managing this destructive pest.

## 1. Introduction

Morphological characteristics are crucial for insect identification and adaptation. The diversity of stripes and body colors, particularly in Lepidoptera and Coleoptera, plays a significant role in courtship, environmental adaptation, and predator avoidance [1,2]. Consequently, these traits are key targets of evolutionary selection [3,4]. Previous research has shed light on the genetic basis of such traits. For instance, Wright [5] discovered that body-color polymorphism in *Drosophila melanogaster* is co-regulated by multiple key enzymes in the pigment synthesis pathway. Similarly, Gompel et al. [6] found that specific wing pigmentation in male *D. melanogaster* is linked to cis-regulatory elements of the yellow gene. In Harmonia axyridis, genome-wide polymorphism studies using ISSR markers revealed that speckle patterns are closely associated with genetic variation and diversity [7]. However, compared to model organisms, the genetic mechanisms regulating body color polymorphism in many economically important beetles remain underexplored.

A prime example of such morphological diversity is *Rhynchophorus ferrugineus* (Coleoptera: Curculionidae), also known as the red palm weevil [8]. This species is primarily recognized as a typical invasive pest that poses a severe threat to palm species such as *Phoenix sylvestris*, *Livistona chinensis*, and *Caryota ochlandra* [9,10]. Due to its devastating impact, it was listed as a quarantine pest by the National Forestry and Grassland Administration in 2005 [11]. Currently, *R. ferrugineus* is distributed primarily in coastal regions and certain inland areas of China, including Sichuan, Chongqing, and Motuo [12]. Beyond its status as a pest, *R. ferrugineus* exhibits striking polymorphism in its pronotum speckle patterns, making it an ideal model for investigating the intersection of pest management and evolutionary genetics.

Regarding the pronotum of *R. ferrugineus*, previous studies have largely focused on descriptive statistics of speckle number and shape, confirming that speckle phenotypes vary significantly across different geographic populations [13,14,15,16]. To further explore the underlying mechanisms of these speckle changes, genome-wide association studies (GWAS) and RNAi technology have emerged as valuable reference approaches [17,18]. GWAS is a powerful, high-throughput approach for detecting genetic variation and screening candidate genes associated with specific traits from the population level [19,20]. Currently, GWAS has been widely applied to dissect complex traits in livestock, agriculture, and human diseases [21,22,23,24,25,26]. It is also extensively utilized in insect polymorphism research; for example, Marina et al. identified 45 core genes related to desiccation resistance in wild *Drosophila* populations using this approach [27]. Despite these technological advances, current research on *R. ferrugineus* predominantly focuses on its life habits, morphological statistics, and comprehensive control [12,13,14,28,29,30,31], leaving its internal genetic mechanisms largely unclear. In this study, we aimed to investigate the molecular mechanisms of speckle variation in *R. ferrugineus* by combining genetic mapping and GWAS to identify key regulatory genes. This work lays a theoretical foundation for understanding the speckle change mechanism and provides new insights into the evolution of insect color traits.

## 2. Materials and Methods

### 2.1. Insect

From June 2020 to July 2021, 189 adult *Rhynchophorus ferrugineus* were collected from damaged *Phoenix canariensis* trunks across Shuangliu District (103°55′ E, 30°34′ N), Xinjin District (103°82′ E, 30°42′ N), and Chongzhou City (103°37′ E, 30°47′ N) in Chengdu City. The collected adults were categorized into 9 distinct phenotypes based on the specific number of distinct black speckles on their pronotum: two-dot (2D), three-dot (3D), four-dot (4D), five-dot (5D), six-dot (6D), seven-dot (7D), eight-dot (8D), and ten-dot (10D) types. Additionally, a mottled type (MD) was defined for individuals exhibiting irregular, extensively merged, or continuous large black patches where individual dots could not be accurately counted. The collected samples were photographed and recorded using a super-resolution microscopy system for insects (Keyence, Osaka, Japan). Representative photos of male and female adults are provided in Supplementary Figure S1. For subsequent artificial propagation, 30 male and 30 female adults were randomly selected, while the remaining individuals were flash-frozen in liquid nitrogen and stored at −80 °C for future analysis. The selected adults were paired (1:1 sex ratio) and reared in polypropylene breeding boxes (21 × 15 × 12 cm) placed in an intelligent artificial climate incubator (Ningbo Jiangnan Instrument Factory, Ningbo, China). The rearing conditions were maintained in constant darkness (0L:24D) at a temperature of 27 ± 1 °C and a relative humidity of 75 ± 5%. Fresh sugarcane (*Saccharum officinarum*) was provided in the breeding boxes to supply moisture and nutrition for the adults and to serve as an oviposition substrate for the females. The sugarcane was replaced every three days. Newly hatched larvae were continuously fed with fresh sugarcane, and coconut shells were provided to facilitate cocooning and pupation [32].

### 2.2. Genetic Population Design

Newly emerged adults without mating with clear stripes and the same quantity of speckles were selected as parents for the inbred group, paired and cultivated as per the same speckle (2D, 3D, 4D, 5D, 6D, 7D, 8D and 10D), female:male = 1:1, and 3 groups were prepared to repeat each type of processing. One male and female newly emerged adult without mating, exhibiting clear stripes and significantly different quantities of speckles (2D, 4D, 8D, and 10D), were selected for the hybrid group [33]. These four specific phenotypes were chosen because they represent the phenotypic extremes and intermediate states, which maximizes the genetic variance for the subsequent quantitative analysis while accommodating the space constraints of indoor artificial breeding. They were paired and cultivated across 6 different combination types. Three groups were prepared to repeat each type of processing, and they were cultivated and bred in the artificial climate box as per the breeding conditions in 1.1. After adult emergence, pronotum speckle number and trait and ranking mode of the F_1_ generation were calculated, recorded and photographed per female and male. Newly emerged adults without mating were selected from inbred and hybrid groups F_1_ generation as per the above inbred/hybrid method to breed the F_2_ generation, and the same was recorded as per the statistical method. After statistics, the adult insects were killed with liquid nitrogen and stored in a −80 °C refrigerator (Haier, Qingdao, China) for later use.

### 2.3. Phenotype Data Collection and Processing

Calculate the phenotype observation data of the inbred and hybrid F_2_ generation female and male individuals, conduct the independent-sample *t* test for each group of phenotype observation data, analyze the inbred group, the hybrid group, and a combined dataset—formed by pooling the phenotypic data of both groups to represent a simulated random mating population—using the quantitative trait mixed inheritance analysis method [34], altogether including 39 models covering one to three pairs of independent major genes, many minor genes, one pair of major gene + polygene, two major genes + polygene, and three major genes + polygene. Use the mixture distribution theoretical basis to calculate the maximum likelihood value of the model and parameter estimate of different generations and principal component distribution; select 5 models with the smallest Akaike information criterion (AIC) and conduct the Kolmogorov–Smirnoff test (_n_W^2^ and D_n_) and fitness test (U1^2^, U2^2^ and U3^2^) to determine the optimum model so as to decide the major genetype attribution and effect separating the individuals from the population [34].

### 2.4. Population Genetic Structure Analysis and Phylogenetic Tree Building Among Individuals

Select 15 pieces of 2D type, 3D type, 4D type, 5D type, 6D type, 8D type, 10D type and MD type, respectively, for 120 pieces in total. Extract genomic DNA from chest tissue of *Rhynchophorus ferrugineus* female and male adults with TruSeq™ Nano DNA Sample Prep kit (Illumina, Shanghai, China) and conduct DNA test. Use the Illumina platform (Illumina, San Diego, CA, USA) to complete library construction of end sequencing, convert the original image data into readable sequential data for quality control, and remove head sequence, sequences not inserted due to intralooping, sequences with 3′ end mass value under 20, sequences with N basic group content exceeding 10%, joint sequences and sequences with mass trimming length of less than 75 bp. Analyze reference nucleotide, variant nucleotide, start site and end site where variation is discovered with reference genome annotation file, conduct functional annotation of gene variations detected in multiple genomes using ANNOVAR (version 20191024) and Genome Analysis Toolkit (GATK, version 4.1), including single-nucleotide polymorphism (SNP) or insertion and deletion (Indel) sequences of small fragments in the genome. Annotate and identify structure variations (SVs) with BreakDancer (version 1.4.5) [35]. Screen out (MAF < 0.05 or deletion rate > 0.5) loci in group SNP, use FastStructure software (version 1.0) to infer group structure. Suppose that the cluster number (K value) of samples is 2~9, respectively; make them clustered and then determine the optimum cluster number as per the cross-validation error (CV error). The K value with minimum cross-validation error corresponds to the optimal cluster number. Then, use the FastTree software (version 2.1.10) based on the p-distance model to build the individual phylogenetic tree [36].

### 2.5. Linkage Disequilibrium Analysis

Calculate the coefficient of association (r^2^) of allelic genes with Haploview(version 4.2) software to determine the LD level of *Rhynchophorus ferrugineus* with different number of speckles. Calculate the average r^2^ value of each distance length, set the maximum distance to 250, and extend the block gradually to determine the optimum maximum distance of each LD block [36]. Finally, map with the empirical method (lod score) and the ranking method and name QTL according to the method proposed by Broman et al. [37].

### 2.6. Genome-Wide Association Study

To effectively control for false positives and systematically compare the associations, we conducted the genome-wide association study (GWAS) using four distinct models implemented in TASSEL software (version 2.1): glm, glmq, mlmk, and mlmkq. The General Linear Models (glm and glmq) evaluate basic associations, with glmq additionally accounting for population structure (Q matrix) to reduce spurious associations. The Mixed Linear Models (mlmk and mlmkq) provide a more stringent analysis by incorporating familial relatedness (Kinship or K matrix) and both Q and K matrices, respectively, to rigorously control for background genetic effects. The Q matrix was calculated with FastStructure, and the group structure was further evaluated by Quantile–Quantile plots mapped with the CMplot package in R software (version 4.2.1) to determine the optimum model [36].

### 2.7. Candidate Gene Prediction

Extract homologous gene information, conduct KEGG annotation and GO function annotation within the candidate interval and analyze molecular function and biological pathways of candidate genes using the biological information database (https://www.kegg.jp/kegg/pathway.html, accessed on 22 March 2026; http://geneontology.org/, accessed on 22 March 2026) on the basis of the population decay level obtained from all models while referring to the genome of *Rhynchophorus ferrugineus* (https://www.ncbi.nlm.nih.gov/genome/?term=Rhynchophorus%20ferrugineus, accessed on 22 March 2026) to further predict candidate genes which might regulate speckle traits.

### 2.8. Candidate Gene Expression Level Analysis

Select 15 pieces of adults of five speckle phenotypes including 2D type, 4D type, 6D type, 8D type and 10D type in residual samples of the same batch of resequencing for quantitative expression level analysis. Use samples that emerged for 1~2d without mating. Extract chest RNA of *Rhynchophorus ferrugineus* with different speckles and synthesize cDNA for qRT-PCR using a LightCycler 480 system (Roche Diagnostics, Mannheim, Germany) and test the relative expression level of candidate genes according to the instructions of the TaKaRa MiniBEST Universal RNA Extraction Kit, PrimeScript™ II 1st Strand cDNA Synthesis Kit and TB Green^®^ Premix Ex Taq™ II kit (Takara, Beijing, China). Repeat 3 times for each sample. Design primers using Primer Select software (version 7.1.0; DNASTAR) (Table 1), take Glyceraldehyde-3-phosphate dehydrogenase (*GAPDH*) as the reference gene [38], and calculate the relative expression level of candidate genes in different speckle phenotypes with the 2^−ΔΔCt^ relative quantitative method.

## 3. Result and Analysis

### 3.1. Analysis of Speckle Genetic Test Results

**Figure 1:**
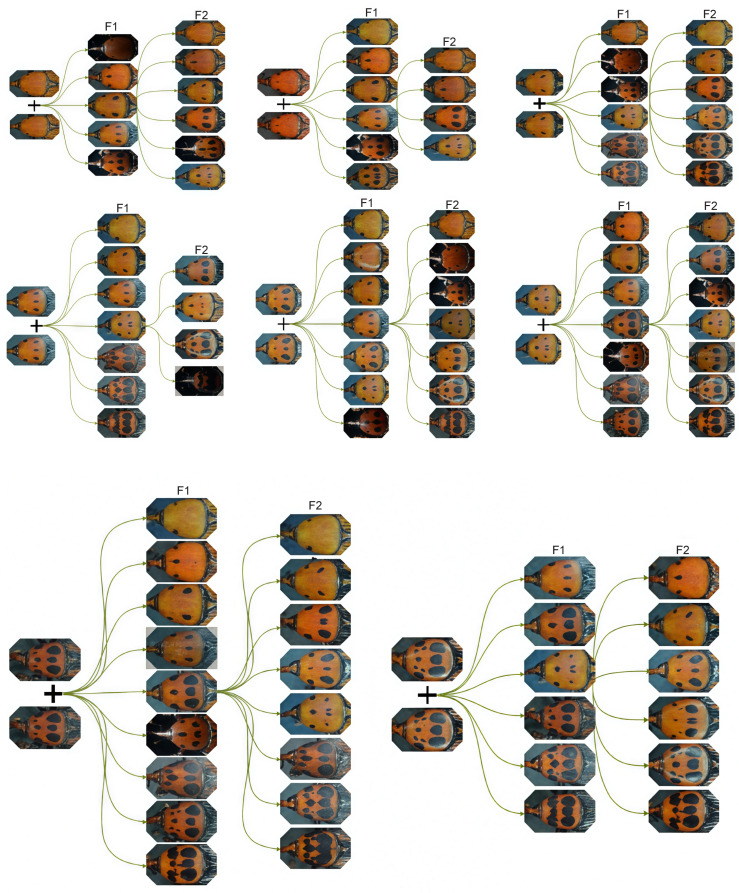
Speckle typing results of inbred group. The images provided are representative photos of the various speckle phenotypes observed, rather than an exhaustive display of all progeny.

**Figure 2:**
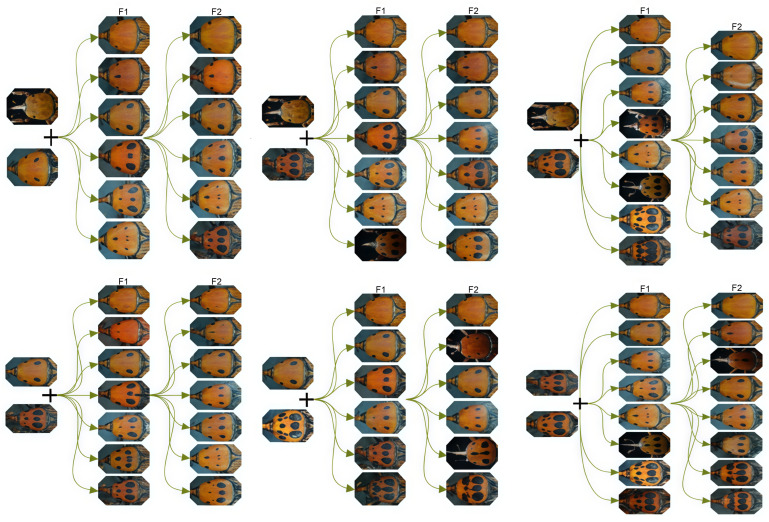
Speckle typing results of hybrid groups. The images provided are representative photos of the various speckle phenotypes observed, rather than an exhaustive display of all progeny.

Based on the phenotypic observation data, an independent-sample *t*-test was conducted to compare the speckle distributions between male and female individuals in the F2 groups. As shown in Table 2 and Table 3, the statistical analysis primarily yielded *p*-values greater than 0.05 (e.g., *p* = 0.37 for the 2D group), indicating no significant difference in the speckle patterns between males and females. Only the 6D type in the inbred population showed a significant difference (*p* < 0.05). In addition, the Pearson correlation coefficient (r) summarizes the concordance of speckle-number distributions between sexes within each group; r values were generally high (>0.80), indicating that male and female distributions followed similar trends. Overall, these results strongly demonstrate that the speckle trait of *R. ferrugineus* adults is a sex-independent quantitative trait. After further statistical analysis of the speckle types, it was discovered that the inbred descendants exhibited significant speckle trait separation (Figure 1 and Figure 2). The progeny displayed a wide, nearly continuous spectrum of phenotypes, and the separation ratio was inconsistent with simple Mendelian segregation. This extensive segregation strongly indicates that the speckle pattern is a quantitative trait controlled by multiple interacting genes, rather than a simple qualitative trait.

Merge the continuous inbred, hybrid and random mating populations into an entirety as a mating population. Conduct a mixed separation analysis for the above three populations according to the observed phenotype value. Among the three mating populations, five potential optimal genetic models were obtained based on the maximum logarithm likelihood function value and the AIC value. However, none of the five models reached a level of significance in the five testing methods (Table 4). According to the AIC value minimum principle (Table 5), the 2MG-EAD model can be deemed as the optimal model for the inbred population and the hybrid population, and the 2MG-CD model can be used as the optimal model for the random mating population. According to error variance, the F_2_ population variance and its component distribution variance, the genetic variance of major genes of the three types of mating F_2_ populations, were 484.9722, 4011.194 and 0, respectively. The major gene inheritability ratios were 99.27%, 98.62% and 0, respectively.

### 3.2. Quality Control Result and Individual Phylogenetic Tree Building

**Figure 3:**
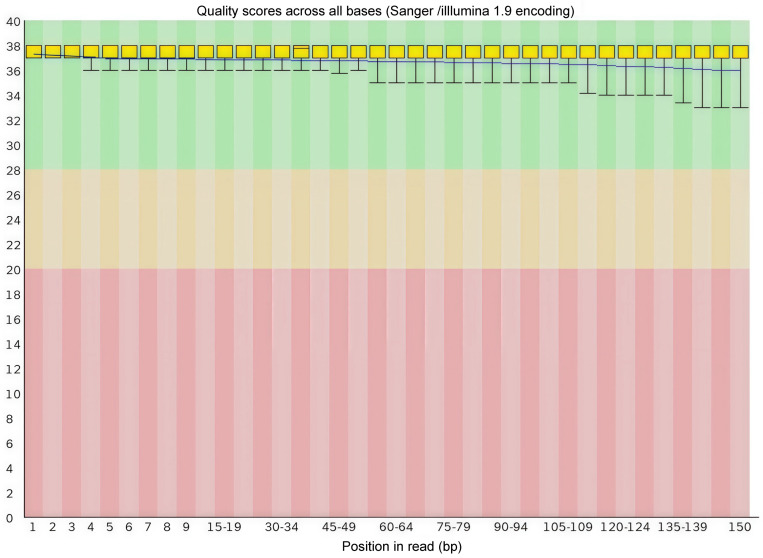
Quality scores across all bases.The yellow boxes represent the inter-quartile range (25th to 75th percentile), and the blue line indicates the mean quality score. The background colors (green, orange, and red) represent calls of very good, reasonable, and poor quality, respectively.

According to DNA agarose gel electrophoresis test of the genomes of the 120 pieces of *Rhynchophorus ferrugineus*, there is a single target band, the average concentration is 121.32 ng/μL, and the OD score is within 1.8~2.29. The DNA is qualified and can be used for cDNA database building and sequencing. After obtaining original data via database building and sequencing, the Trimmomatic result is used for quality analysis. All base Qphred values are higher than 30 and base accuracy reaches as high as 99.9% (Figure 3). The sequencing error rate is below 0.04% and the total error rate is 0.03%. The sequencing results and the quality of the samples were good and can thereby be used for further research and analysis.

**Figure 4:**
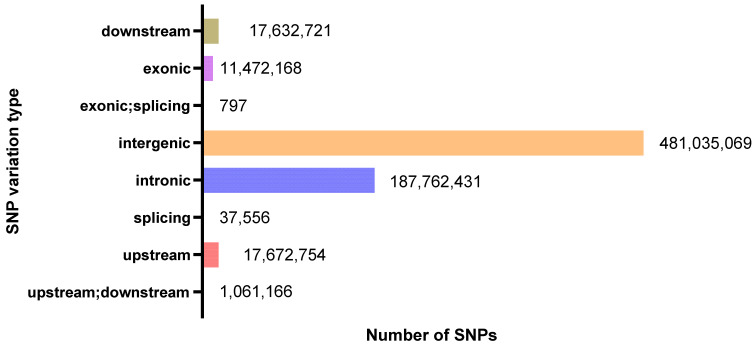
SNP annotation results.

**Figure 5:**
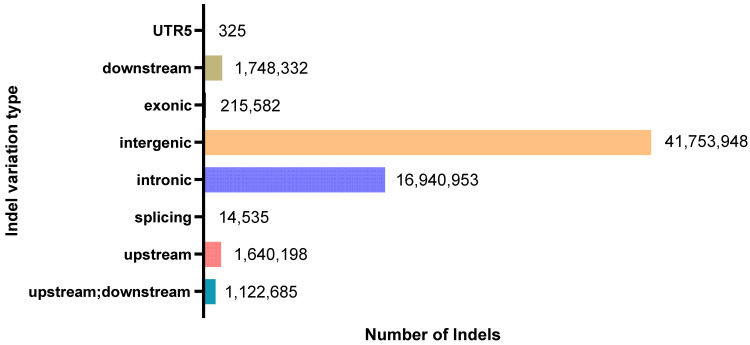
Indel annotation results.

After rigorous SNP calling and filtering using the Genome Analysis Toolkit (GATK, version 4.1), a total of 11,377,676 unique, high-quality SNPs were identified across the 120 *Rhynchophorus ferrugineus* individuals. For the functional annotation using Ensembl-VEP, we calculated the cumulative instances of these variants across all sequenced individuals to reflect the population-level variant burden, resulting in a total of 716,674,662 cumulative SNP occurrences (Figure 4). Among these occurrences, 17,672,754 were within the 2 kb region upstream of genes, 17,632,721 were within the 2 kb region downstream of genes, 11,472,168 were within the exon region, 187,762,431 were within the intron region, and 481,035,069 were within the intergenic region. Furthermore, there were 4,024,596 nonsynonymous mutations and 7,117,716 synonymous mutations. Additionally, 86,303 mutations resulted in stop-gains, 15,809 resulted in stop-losses, and there are 228,541 unknown mutations. Altogether, 215,582 InDels have been obtained, including 209,913 InDels which can be compared to the genomic library. Due to incomplete ORF of genes in the annotation file, 11,146,373 InDels are newly annotated. Through annotation of InDels of *Rhynchophorus ferrugineus* with ANNOVAR software, there are 1,640,198 InDel mutations within the 2 kb region upstream of genes and 215,582 InDel mutations within the exon region, where 979 InDel mutations result in translation termination generation and 240 InDel mutations result in translation termination loss. There are 126,506 frameshift mutation InDel mutations and 16,770 non-frameshift mutation InDel mutations. There are 16,940,953 InDel mutations within the intron region, 14,535 InDel mutations within the splicing locus region and 1,748,332 InDel mutations within the 2 kb region downstream of genes (Figure 5).

**Figure 6:**
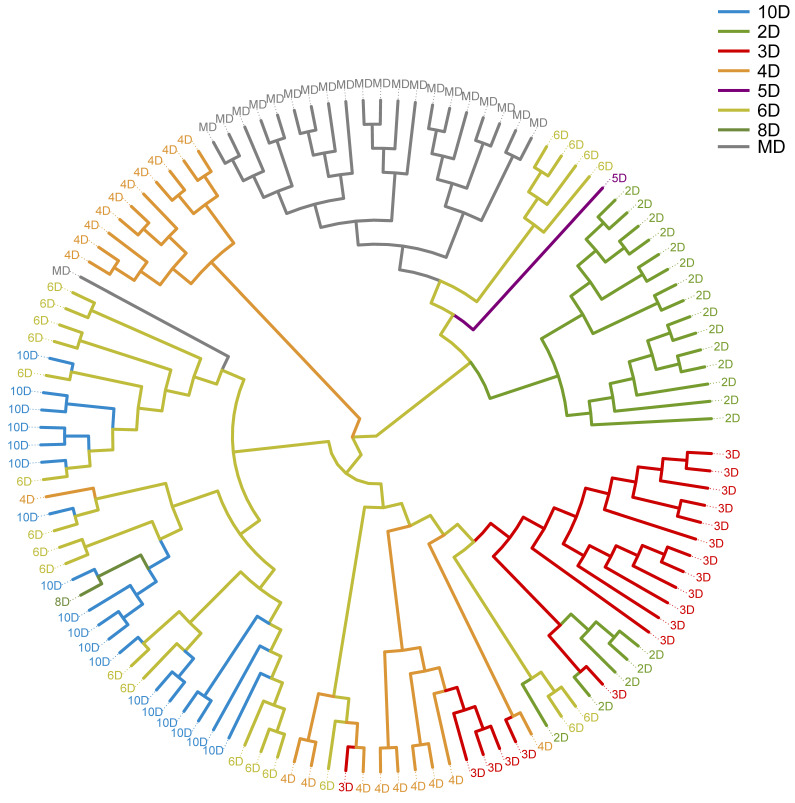
Phylogenetic tree of SNP and speckle phenotype.

The phylogenetic tree was constructed based on genome-wide SNP distance of 120 samples to visualize the overall genetic similarity among individuals (Figure 6). In the figure, different colors represent different observed speckle phenotypes. Most speckle phenotypes can be clustered into relatively close branches, with only a few overlapping phenotype values. The clustering pattern suggests that several phenotype types (such as 2D, 3D, 4D, 5D and MD) tend to group together, while some types show partial overlap (for example, 6D and 10D). It should be noted that this tree is mainly used to display the broad clustering trend of the samples and the relationship between genetic similarity and phenotype categories, rather than to make strict phylogenetic inference or subspecies-level conclusions.

### 3.3. Population Structure Analysis and Linkage Disequilibrium Analysis

**Figure 7:**
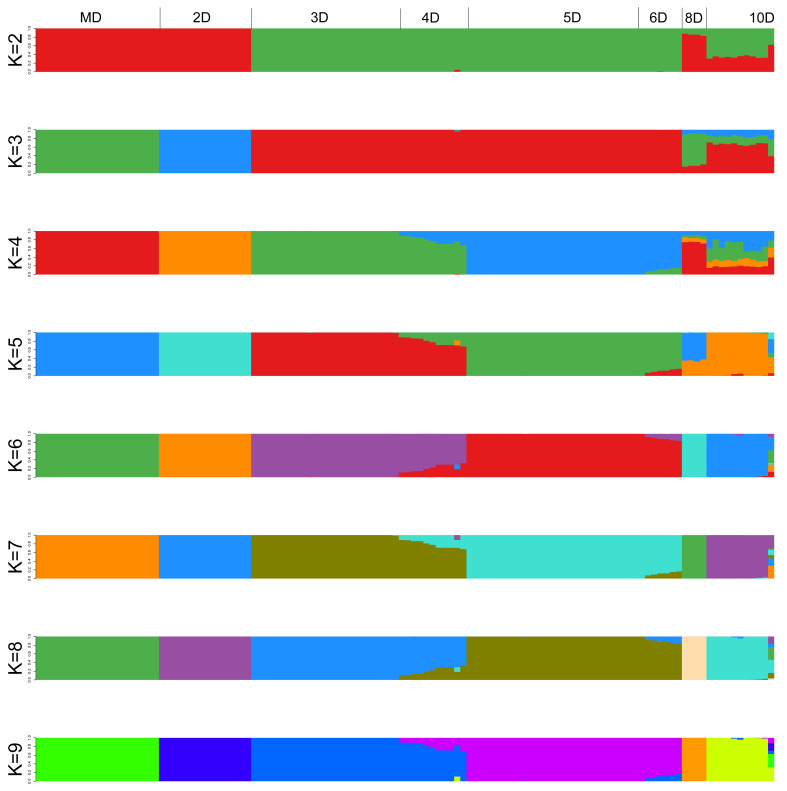
Population genetic structure of the 120 *Rhynchophorus ferrugineus* individuals. The population structure map was generated using FastStructure. Each vertical bar represents a single individual. The different colors within each bar indicate the estimated admixture proportions of different theoretical ancestral subpopulations for that individual. Based on the cross-validation error, the optimal number of clusters was determined to be K = 7, indicating the presence of seven distinct genetic subpopulations within the sampled cohort. Black vertical lines separate the major phenotype groups.

**Figure 8:**
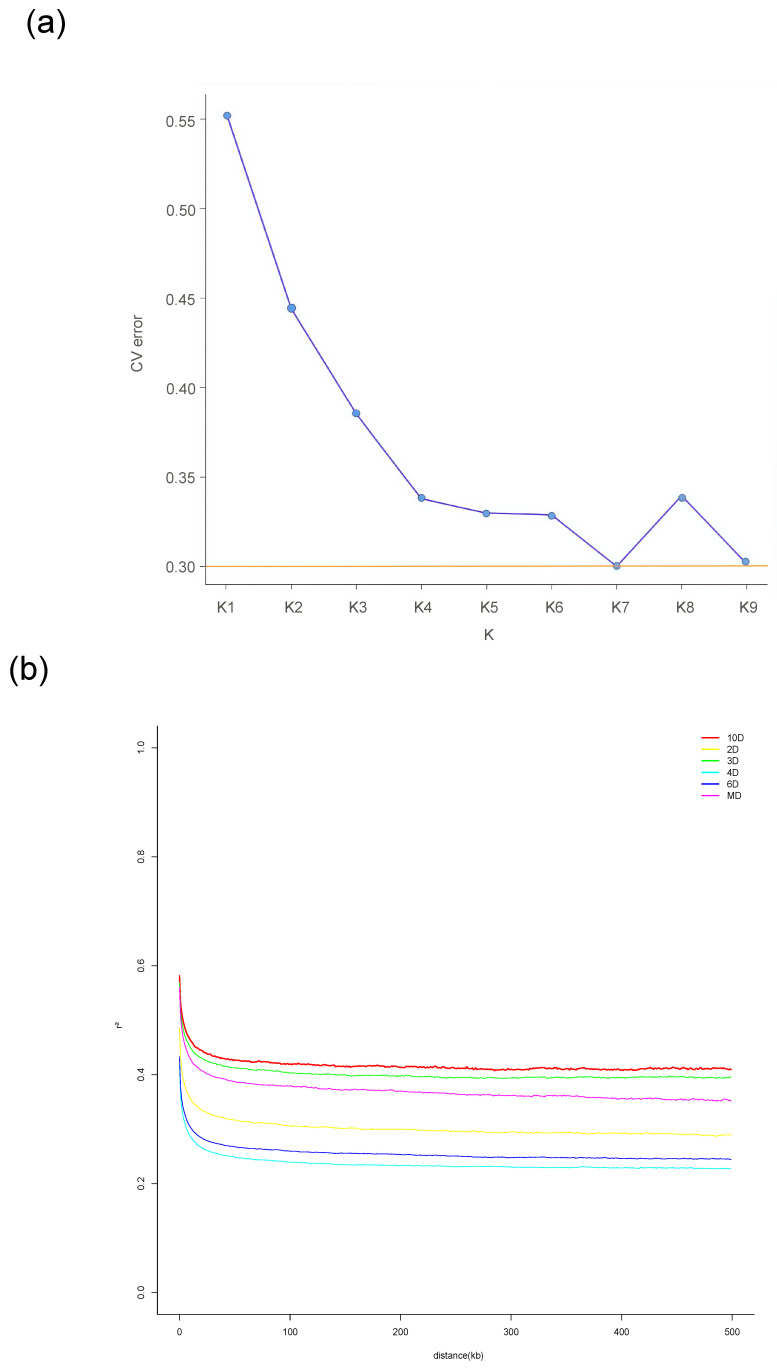
(**a**): The cross-validation (CV) error plot for different K values. The blue line and dots represent the CV error values for each K. The yellow line highlights the minimum CV error, indicating the optimal number of clusters. (**b**) Linkage disequilibrium (LD) decay plot. The X-axis represents the physical distance across the genome (in kb), and the Y-axis represents the squared allele frequency correlation (r^2^). The different colored lines represent the LD decay trends for the six groups (10D, 2D, 3D, 4D, 6D, and MD).

A total of 20,000 SNPs with top LD scores and excellent polymorphism are selected to map the population genetic structure chart within K = 2~9 (Figure 7). The result shows that *Rhynchophorus ferrugineus* notum speckles can be divided into seven subpopulations (Figure 8a, K = 7). The r^2^ value of all SNPs within 500 Mb in 120 samples is analyzed and LD decay trend is determined. To prevent the impact of domestication caused by artificial propagation on its decay distance and rate, the decay distance of 500 kb corresponding to stable r^2^ is finally selected as the interval distance for the next candidate gene prediction (Figure 8b). At last, 19 QTLs related to speckles have been obtained (Table 6).

### 3.4. Genome-Wide Association Studies Result

**Figure 9:**
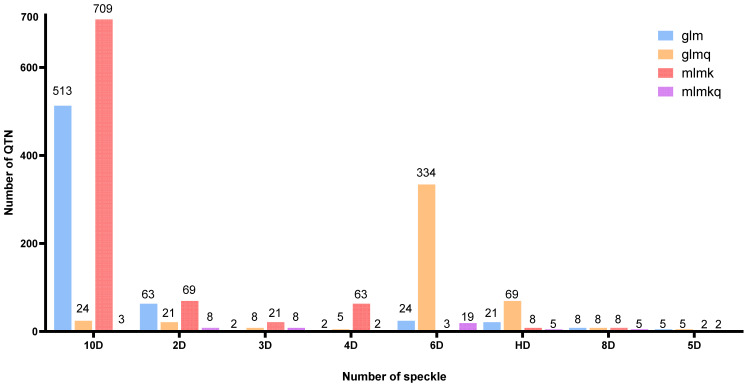
QTN detected by different models.

**Figure 10:**
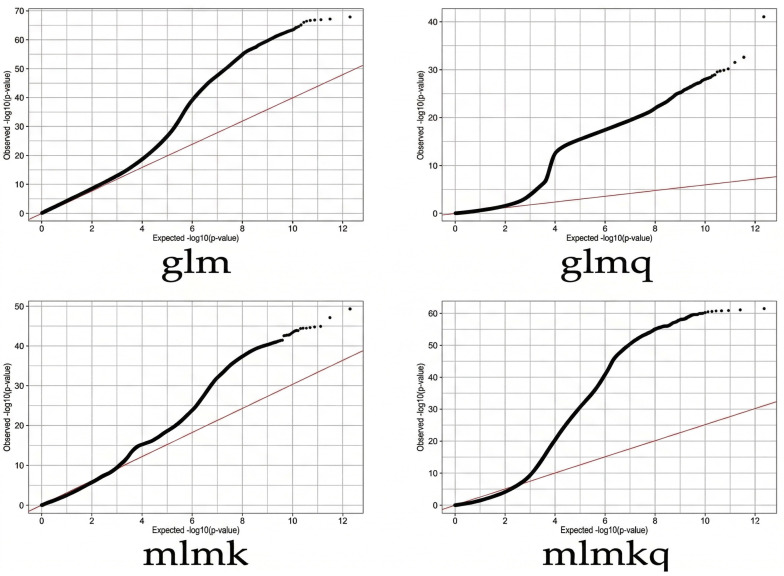
Q-Q diagrams for four models.The straight red diagonal line represents the expected distribution of *p*-values under the null hypothesis (no association). The scattered black dots represent the observed *p*-values. The deviation of the observed values from the red line at the upper right indicates significant associations.

GWASs have been conducted for 120 different speckle phenotype traits of *Rhynchophorus ferrugineus* using four methods, including glm, glmq, mlmk and mlmkq, and 638, 474, 883 and 52 significant genes (QTN) are detected related to speckle trait (Figure 9). Association analysis is conducted according to phenotype traits and genetic variation data to obtain the *p* value and to map the Q-Q chart (Figure 10). The *p* values of SNP markers detected by glmq and mlmkq models deviate from expected *p* values, indicating a significant false positive. The *p* values of SNP markers detected by glm and mlmk models are basically consistent with expected *p* values, indicating relatively reliable significant locus results detected by glm and mlmk models. To fully explore genotype and appearance trait data, this study uses four models to analyze relevant candidate genes jointly obtained from eight speckle phenotypes.

**Figure 11:**
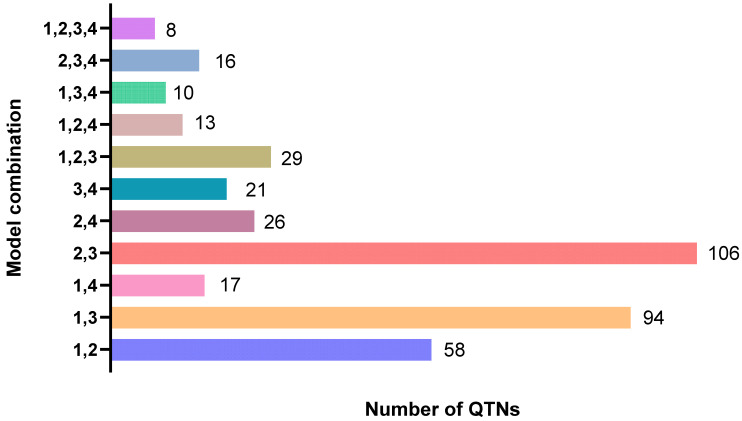
QTN jointly detected by different methods.

After detecting significant SNPs in different speckle types using different GWAS methods, it is discovered that eight highly correlated SNPs are jointly detected by the four models (Table 7). When ranking and combining different association analysis methods to analyze SNP results, it is found that significant SNPs are detected with two methods, which are 58, 94, 17, 106, 26, and 21, respectively (Figure 11). To further narrow down the search scope, 78 significant SNPs were jointly detected with three methods, with 29, 13, 10, and 16 respectively, and eight SNPs were jointly detected with four methods. According to the above study, among the four methods, more SNPs can generally be detected with glm, glmq and mlmk methods. As for different ranking and combining of two methods, the most SNPs are detected with the glmq and mlmk combination.

### 3.5. Result from Combining Linkage Analysis with Association Analysis

By combining linkage analysis with a single GWAS association analysis and comparing mapped QTL/QTNs with two analysis methods for marker positions, it is finally discovered that 12 QTNs are within the QTL interval mapped by linkage analysis (Table 8). Therefore, the 12 QTNs are used as the study objects of the next stage.

### 3.6. Determining Potential Candidate Genes Combining Linkage Analysis with GWAS

**Figure 12:**
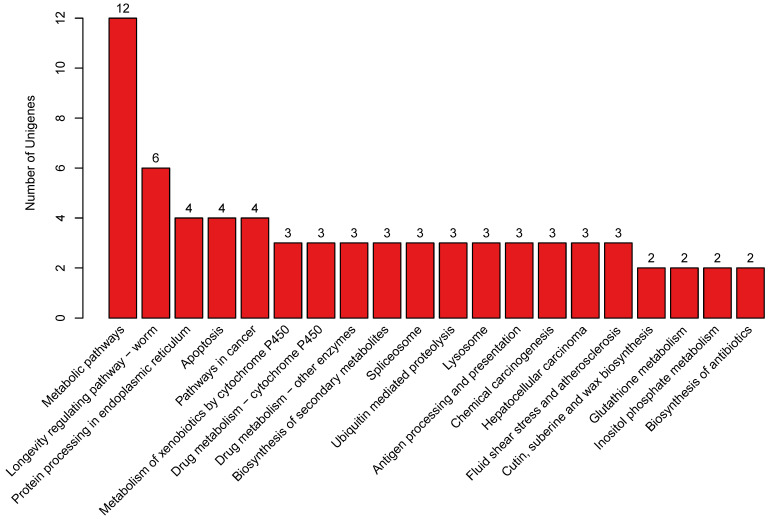
Annotation pathways of 22 genes in KEGG.

**Figure 13:**
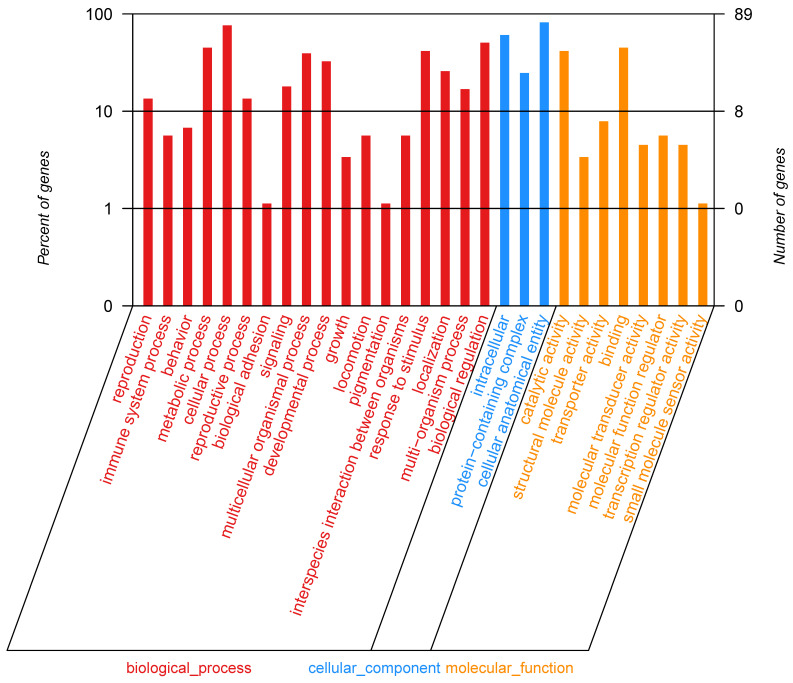
Annotation pathways of 22 genes in GO.

According to the contrastive analysis of candidate genes obtained through linkage analysis and GWAS, it is discovered that LD decay transitions to a steady state after 500 kb (Figure 8). Therefore, 250 kb of physical distance is deemed as an interval and possible candidate genes are searched on both sides of the QTN to obtain potential candidate genes taking the GWAS result into consideration. Finally, 22 genes are discovered within the interval. When searching for potential candidate genes with linkage analysis and GWAS analysis, as for expression information annotated in KEGG and GO databases, attention is paid to genes related to pigmentation, signal transduction, and metabolic processes. Ultimately, it is found that three genes may be involved in the pigment synthesis pathway (Figure 12 and Figure 13). Based on the annotation information of these genes and their functions in metabolic pathways, it is preliminarily determined that the three genes are highly associated with speckle changes (Table 9).

### 3.7. Analysis of Expression Level of Candidate Genes

**Figure 14:**
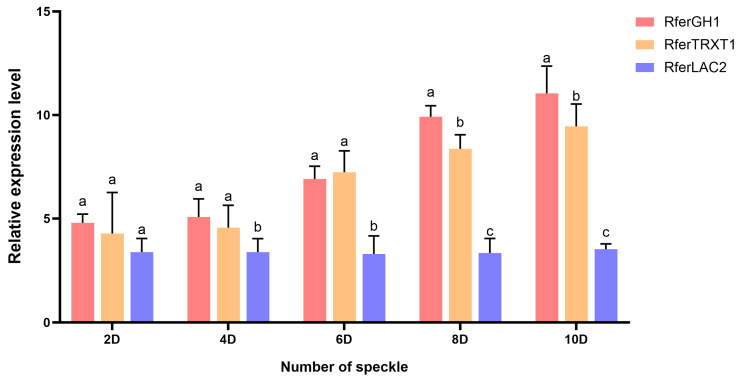
Relative expression levels of three candidate genes across five speckle phenotypes. The data are presented as the mean ± standard deviation (SD) of three biological replicates. Different lowercase letters above the bars indicate significant differences in the relative expression level of the same gene across different speckle phenotypes (*p* < 0.05, one-way ANOVA).

The expression levels of *RferGH1* and *RferTRXR1* varied significantly among the five speckle phenotypes. In contrast, the expression level of *RferLAC2* did not exhibit significant differences across the different speckle types. Among the five types of speckle samples, the expression level of *RferGH1* and *RferTRXR1* exhibit an overall increasing trend, with small changes in the 2D and 4D types, and a rising trend in the 6D, 8D, and 10D types, indicating that they play a stronger controlling role in the formation of a high number of speckle types. Changes in the expression levels of *RferGH1* and *RferTRXR1* are quite similar to the speckle number change increasing trend and the expression level is the highest in the 10D-type speckle (Figure 14). The detection of the expression levels of the two candidate genes reveals that the expression level in the speckle type with more speckles is significantly higher than that in the speckle type with less speckles, indicating that *RferGH1* and *RferTRXR1* genes may have a positive regulatory effect on the number of speckle number traits of *Rhynchophorus ferrugineus*.

## 4. Discussion

In the natural world, diversified speckles and body colors of species are the most important phenotype change traits [31]. The speckles of the pronotum of *Rhynchophorus ferrugineus* are highly complicated black dot patterns, which can be used as a good speckle study object [39]. As for quantitative trait separation analysis, a segregative generation population built through genetic experiments is used to predict its genetic model and major gene genotype of an individual or family through the observed phenotype value [34]. The method is reported to be widely used in quantitative trait genetic analysis of plants and animals [40,41,42]. It is discovered in the study that speckle regulation of *Rhynchophorus ferrugineus* is controlled by two major genes and expressed as additive effect—dominance effect. According to analysis of Hua Liu et al. [43], Phoma arachidicola Marasas Pauer & Boerema resistance has shown genetic rules of two multi-generational populations with significant differences. The result has shown that Phoma arachidicola Marasas Pauer & Boerema resistance is controlled by two pairs of additive–dominance–epistatic major gene + additive–dominance polygenes.

When analyzing complex traits, different analysis models are used to try to explore all potential QTN [44,45,46] related to target traits to avoid the risk of potentially high false-positive rates. Liu et al. [47] has conducted genome-wide association studies for early round-grain nonglutinous rice based on general linear model and screened candidate genes related to zinc content of kernels. Altogether, 12 significant SNPs are correlated, which are distributed on chromosomes 1, 2, 4, 10, and 11. Zeng et al. [48] have screened new Single Nucleotide polymorphism (SNP) and Quantitative Trait Locus (QTL) related to halotolerance from *Glycine max* using the General Linear Model (GLM) and the Mixed Linear Model (MLM), and finally identified significant SNP markers on chromosomes 2, 3, 14, 16 and 20, providing the basis for breeding halotolerant varieties in *Glycine max* parent selection and germplasm evaluation. The study has mapped QTLs of speckle traits of *Rhynchophorus ferrugineus* with GLM and MLM while taking into consideration of four types of multilocus correlation analysis methods including glm, glmq, mlmk and mlmkq. Eight significant QTNs regulating speckle traits of *Rhynchophorus ferrugineus* are obtained with four types of multilocus genome-wide association studies and 12 traits related QTNs are further discovered while taking into consideration of linkage analysis and different association studies.

Three potential candidate genes (*RferGH1*, *RferTRXR1* and *RferLAC2*) are found through many analysis methods; GWAS and bioinformatics analysis and are further verified through Fluorescence Quantitative PCR technology. It is finally confirmed that *RferGH1* and *RferTRXR1* are genes related to speckle change formation of *Rhynchophorus ferrugineus,* and they are Glutathione hydrolase (GH) and Thioredoxin reductase (TrxR). Glutathione is an important component that maintains intracellular redox balance and regulates the production of cell melanin through various mechanisms. The metabolism of thiol compounds such as glutathione is closely related to the synthesis of melanin, especially the relative synthesis of eumelanin and pheomelanin [49,50]. Glutathione hydrolase can hydrolyze glutathione into glutamic acid, cysteine, and glycine, where cysteine, as a type of sulfhydryl compound, is discovered to be capable of inhibiting cell eumelanin generation more safely and effectively [49]. Thioredoxin reductase belongs to the pyridine nucleotide disulfide oxidoreductase family and forms the Trx system together with thioredoxin (Trx) and nicotinamide adenine dinucleotide phosphate (NADPH). It plays an important role in maintaining intracellular redox homeostasis and protecting organisms from oxidative damage [51,52]. TrxR was first discovered in Escherichia coli and is the major enzyme in Escherichia coli that reduces ribonucleotides to deoxyribonucleotides [53]. Currently, the study on TrxR is mainly focusing on pathogenesis of human diseases, the stress resistance of plants, etc. [54,55]. Rozell et al. prepared fluorescent antibodies from TrxR and Trx separated from rat livers, observed TrxR and Trx cell and tissue distribution within rat bodies and discovered that there were high levels of TrxR and Trx in melanophores and Langerhans cells of rat skin and relatively low levels in cells formed by stratum granulosum and stratum corneum [56]. Schallreuter et al. [57] discovered that the activity and concentration of thioredoxin reductase/thioredoxin can qualitatively or quantitatively regulate the melanin synthesis pathway. The gene can control speckle traits by affecting quantitative expression of melanin directly [58,59]. Therefore, we can predict that *RferGH1* and *RferTRXR1* can regulate the melanin synthesis pathway by adjusting the presence and activity of glutathione and thioredoxin qualitatively or quantitatively, so as to control speckle trait change in *Rhynchophorus ferrugineus.*

In conclusion, the study has explored candidate genes related to speckle traits of *Rhynchophorus ferrugineus* using traditional genetic tests and modern molecular biology techniques as well as multilocus GWAS, linkage disequilibrium analysis and bioinformatics analysis, and confirmed two candidate genes that may be related to speckle change in *Rhynchophorus ferrugineus* preliminarily (*RferGH1* and *RferTRXR1*), which has laid the theoretical basis for further exploring the cause of speckle polymorphism of *Rhynchophorus ferrugineus*, the speckle change mechanism of *Rhynchophorus ferrugineus*, and enriching the insect body color and speckle polymorphism formation mechanism.

Interestingly, while *RferGH1* and *RferTRXR1* exhibited expression levels that positively correlated with speckle number, *RferLAC2* did not show significant differences across the phenotypes. This divergence can be explained by their respective roles in the insect melanin and sclerotization pathways. *LAC2* (Laccase 2) is a highly conserved, foundational enzyme essential for general cuticle tanning and sclerotization immediately following adult emergence. Because all *R. ferrugineus* adults require extensive cuticle hardening regardless of their specific dot patterns, *RferLAC2* is likely constitutively expressed at high levels across all individuals. In contrast, *RferGH1* and *RferTRXR1* appear to act as rate-limiting regulators specifically recruited for the localized overproduction of melanin that forms the distinct dark speckles, thereby displaying phenotype-dependent expression.

## 5. Conclusions

In conclusion, this study elucidates the genetic architecture and molecular mechanisms underlying pronotum speckle variation in the invasive pest *Rhynchophorus ferrugineus*. Through rigorous genetic crosses and quantitative trait mixed analysis, we demonstrated that speckle polymorphism is a sex-independent quantitative trait primarily governed by major loci exhibiting additive and dominance effects. By integrating multi-locus GWAS with linkage analysis, we successfully mapped key genomic regions associated with this trait. Subsequent bioinformatics and expression analyses identified *RferGH1* and *RferTRXR1*—genes involved in the melanin synthesis pathway—as core regulators, both of which exhibited significantly higher expression in individuals with greater speckle numbers. These findings not only advance our fundamental understanding of the evolutionary genetics and molecular basis of insect pigmentation but also provide valuable genetic markers that could be utilized for the monitoring and management of this destructive palm pest.

## Figures and Tables

**Table 1:** Fluorescence quantitative primer sequence.

Primer Name	Primer Sequence
RferLAC2-qF	5′-ACCGATATTCGCCACCATTAC-3′
RferLAC2-qR	5′-ATTGTCTGGATCAAGACCCTC-3′
RferGH1-qF	5′-CGCTTCTGGTAACATTATCCCAT-3′
RferGH1-qR	5′-ATGTTATTTTCGACGATACAGAAGTT-3′
RferTRXR1-qF	5′-CGCCTTCTGGTAATTCTATCGCCAT-3′
RferTRXR1-qR	5′-CGCTTCTGGTAACATTCTATCCCAT-3′
RferGAPDH-F	5′-CGCTTCTGGTAACATTATCCAT-3′
RferGAPDH-R	5′-CGTCGACAACGGCAACATGAC-3′

**Table 2:** Descriptive statistics, Pearson correlation (r), and independent-sample *t*-test (*p*) of speckle number between male and female adults in inbred F2 groups.

Group	Female /Male	Mean	Standard Deviation	Variance	Skewness	Kurtosis	Correlation Coefficient (Pearson r)	*t*-Test *p*-Value
2D	♂	4.67	11.78	138.75	2.97	8.87	0.99	0.37
	♀	5.22	13.43	180.44	2.99	8.94		
3D	♂	5.67	8.41	70.75	2.11	4.80	0.84	0.07
	♀	10.67	12.44	154.75	1.09	−0.24		
4D	♂	6.33	11.57	133.75	1.89	2.56	0.91	0.45
	♀	9.00	19.44	378.00	2.83	8.16		
5D	♂	4.44	8.22	67.53	2.82	8.18	0.82	0.60
	♀	5.33	7.75	60.00	1.30	0.52		
6D	♂	6.89	10.08	101.61	2.68	7.60	0.97	0.03 *
	♀	9.44	11.59	134.28	2.51	6.95		
7D	♂	5.22	7.05	49.69	1.67	2.55	0.88	0.70
	♀	4.78	6.76	45.69	2.51	6.88		
8D	♂	7.89	11.52	132.61	2.50	6.47	0.90	0.12
	♀	11.00	12.23	149.50	1.77	3.13		
10D	♂	8.56	12.95	167.78	2.01	3.87	0.91	0.69
	♀	9.33	13.48	181.75	2.60	7.29		

Note: Pearson r indicates the concordance of speckle-number distributions between sexes within each group. The *t*-test *p*-value is from an independent-sample *t*-test comparing male vs. female speckle numbers. * *p* < 0.05 indicates a significant sex difference.

**Table 3:** Descriptive statistics, Pearson correlation (r), and independent-sample *t*-test (*p*) of speckle number between male and female adults in hybrid F2 groups.

Group	Female/Male	Mean	Standard Deviation	Variance	Skewness	Correlation Coefficient (Pearson r)	*t*-Test *p*-Value
2D × 4D	♂	15.00	23.44	549.50	1.49	0.97	0.12
	♀	22.44	34.49	1189.28	1.65		
2D × 8D	♂	15.44	16.90	285.78	1.48	0.97	0.10
	♀	27.78	35.56	1264.19	1.68		
2D × 10D	♂	8.89	7.62	58.11	0.93	0.83	0.67
	♀	8.22	7.58	57.44	1.38		
4D × 8D	♂	13.78	15.17	230.19	0.93	0.98	0.22
	♀	17.67	23.09	533.00	1.23		
4D × 10D	♂	8.56	13.06	170.53	1.84	0.97	0.87
	♀	8.78	10.73	115.19	1.85		
8D × 10D	♂	18.22	18.20	331.19	0.79	0.90	0.88
	♀	17.78	19.45	378.44	1.31		

Note: Pearson r indicates the concordance of speckle-number distributions between sexes within each group. The *t*-test *p*-value is from an independent-sample *t*-test comparing male vs. female speckle numbers. *p* < 0.05 indicates a significant sex difference.

**Table 4:** Statistical results of posterior probability in random population.

Group	Model	Homogeneity Test			Smirnov Test _n_W^2^	Kolmogorov Test D_n_
		U1^2^	U2^2^	U3^2^		
Inbred	2MG-CD	0.9479	0.8889	0.7600	0.9555	0.9216
	2MG-EAD	0.9998	0.9892	0.9577	0.9071	0.9277
	MX2-EA-AD	0.9855	0.9763	0.9615	0.8982	0.8973
	MX2-A-AD	0.2776	0.4756	0.1766	0.2582	0.1740
	MX2-AD-AD	0.9948	0.9866	0.9667	0.9051	0.9189
Hybrid	2MG-EAD	0.7245	0.9413	0.2844	0.8312	0.9296
	1MG-AD	0.9730	0.9535	0.7158	0.9769	0.9754
	1MG-EAD	0.9862	0.9446	0.8326	0.9946	0.9949
	1MG-NCD	0.3206	0.5712	0.1137	0.2663	0.3075
	2MG-EA	0.9546	0.8833	0.7136	0.9745	0.9716
Random	2MG-CD	0.5659	0.6631	0.6306	0.8048	0.8074
	2MG-EAD	0.9943	0.9023	0.6434	0.9716	0.9769
	MX2-EA-AD	0.4009	0.4419	0.8591	0.6307	0.8118
	MX2-A-AD	0.5045	0.5662	0.7717	0.7706	0.9062
	MX2-AD-AD	0.9726	0.9104	0.7510	0.9763	0.9610

**Table 5:** Result of mixed separation analysis in random population.

Group	Model	Max Likelihood Value	AIC Value AIC	MG-Genetic Variance MG-Var	MG-Heritability (%)
Inbred	2MG-EAD	−114.9168	237.8335	484.9722	99.2722
	1MG-NCD	−119.9981	249.9963	484.9722	99.2722
	1MG-EAD	−120.0112	250.0225	484.9722	99.2722
	2MG-EA	−121.671	251.3421	484.9722	99.2722
	1MG-AD	−119.9806	251.9612	484.9722	99.2722
Hybrid	2MG-EAD	−159.1632	326.3264	4011.194	98.6231
	1MG-NCD	−159.2551	328.5101	4011.194	98.6231
	1MG-AD	−158.7134	329.4267	4011.194	98.6231
	2MG-EA	−161.567	331.134	4011.194	98.6231
	1MG-EAD	−160.5901	331.1803	4011.194	98.6231
Random	2MG-CD	−135.6873	281.3746	0	0
	2MG-EAD	−143.1431	294.2862	3505.683	53.506
	MX2-EA-AD	−146.7755	297.5511	2683.43	40.9562
	MX2-A-AD	−146.2925	298.585	2875.761	43.8917
	MX2-AD-AD	−145.588	301.176	3039.191	46.3861

**Table 6:** Statistical results of posterior probability in QTL.

QTL Name	Method	Speckle	Left Position	Right Position	LOD Score	PVE Score	ADD1	ADD2	ADD3	ADD4
Q-Rf-2-2	lod3	10D	GM_474026	GM_471089	6.79	9.86	1.26	−0.86	1.51	−2.23
Q-Rf-4-3	lod3	10D	GM_124027	GM_121090	4.57	10.31	−1.19	−0.98	0.98	1.23
Q-Rf-5-4	lod3	2D	GM_354028	GM_411567	4.41	8.60	0.81	1.01	−1.19	−0.88
Q-Rf-6-2	lod3	3D	GM_440729	GM_481092	4.29	7.25	−0.14	1.11	−0.34	−0.46
Q-Rf-4-6	lod3	4D	GM_132330	GM_156734	5.39	4.87	−0.92	0.89	−0.35	0.37
Q-Rf-8-7	lod3	6D	GM_304564	GM_317109	5.23	5.05	−0.45	1.42	−0.19	−0.78
Q-Rf-1-10	lod3	MD	GM_185403	GM_189671	6.24	5.14	0.97	−0.87	1.01	−1.52
Q-Rf-2-9	lod3	3D	GM_120303	GM_124752	3.35	6.34	−0.63	1.11	−0.10	−0.77
Q-Rf-4-5	Ranking	4D	GM_154034	GM_171097	5.21	4.61	0.41	−0.49	1.01	−1.15
Q-Rf-2-11	lod3	MD	GM_454003	GM_465010	3.58	8.22	−0.18	0.65	1.21	−0.46
Q-Rf-6-8	lod3	6D	GM_325947	GM_365784	4.49	4.72	0.89	0.59	0.60	−1.08
Q-Rf-2-4	lod3	8D	GM_164322	GM_168493	5.57	5.35	1.07	−0.23	−1.83	−0.01
Q-Rf-7-10	Ranking	5D	GM_446742	GM_449887	6.42	5.03	0.56	−0.56	0.49	−1.12
Q-Rf-3-13	lod3	2D	GM_278039	GM_301102	5.43	7.92	1.23	−0.47	0.67	−0.68
Q-Rf-14-4	lod3	3D	GM_321626	GM_345098	4.56	6.57	0.42	−2.00	−0.21	−0.43
Q-Rf-9-2	lod3	10D	GM_178041	GM_198461	6.57	5.09	0.66	−0.97	1.45	0.27
Q-Rf-4-11	lod3	2D	GM_284735	GM_285923	6.41	8.58	0.45	−1.20	1.09	−0.23
Q-Rf-8-12	lod3	4D	GM_361843	GM_364616	4.29	5.62	−0.37	−0.67	0.30	0.87
Q-Rf-13-5	lod3	MD	GM_428572	GM_430899	4.39	6.47	0.09	0.32	0.65	−0.59

**Table 7:** Associated QTNs detected by four models.

Method	Marker	Marker Position	SNP Number	QTN Effect	LOD Score	Phenotype Contribution Rate r^2^ (%)
Mlmk, glm	*RferPSDX6*	10,014,549	191,059	0.43	3.98, 5.96	3.28, 5.83
mlmk, glmq	*RferDDR1*	10,000,408	125,779	−1.30	3.41, 4.09	3.38, 5.22
mlm, glmq	*RferMAPK11*	10,014,584	217,729	−2.2 × 10^−0.5^	4.20, 4.26	3.72, 4.88
glm, glmq	*RferKLN*	10,000,002	198,098	0.36	4.16, 5.13	3.25, 4.70
glm, mlmk	*RferMAPK1*	10,014,526	61,472	0.52	2.98, 4.10	2.74, 3.21
glmq, mlmq	*RferPY*	10,000,001	176,798	−0.39	3.80, 4.54	2.82, 3.16
mlm, glmq	*RferNAT2*	10,000,014	76,478	−0.49	2.06, 3.68	1.83, 2.46
glm, glmq	*RferSFMBT1*	10,000,078	139,910	0.29	4.62, 4.83	1.92, 2.77

**Table 8:** Mapping of speckle-related genes through linkage and association analysis.

QTN Name	Method 1	Method 2	Marker Position	QTN Effect	LOD Score	Phenotype Contribution Rate (%) r^2^ (%)
*RferGH1*	mlmk	lod3	401,077–427,946	0.81	6.53	3.84
*RferGRL*	glm	Ranking	1,636,749–1,657,369	1.22 × 10^−0.5^	4.41	3.25
*RferLAC2*	glm	lod3	1,178,945–1,210,902	0.70	3.49	4.14
*RferANKDR50*	mlmkq	lod3	81,434–98,424	−0.89	6.19	3.54
*RferXPO5*	mlmk	lod3	1,384,686–1,395,915	−0.82	5.25	4.61
*RferUP1*	glmq	lod3	304,883–322,655	0.37	5.28	5.23
*RferTRXR1*	mlmk	lod3	40,721–42,259	0.85	3.35	7.75
*RferTNPO3*	glm	lod3	608,721–617,890	−1.00 × 10^−0.3^	6.21	4.85
*RferTBC1*	mlmk	Ranking	587,270–598,384	−0.65	3.88	5.60
*RferUP2*	glmq	lod3	70,476–84,976	−0.67	4.79	3.92
*RferCDH1*	mlmkq	lod3	165,785–283,814	0.85	5.57	4.57
*RferLAC4*	glm	lod3	1,447,348–1,475,308	−0.60	3.62	3.25

Note: LOD Score (logarithm of the odds) indicates the statistical significance of the QTN-trait association. The phenotype contribution rate (PVE%) represents the proportion of total phenotypic variance explained by this specific genetic locus; r^2^ (%) reflects the linkage disequilibrium level.

**Table 9:** Mapping of speckle related genes by association analysis and linkage analysis.

Gene	K Number	Position (bp)	KEGG Annotation	GO Annotation
*RferGH1*	K13728	401,077– 427,946	DCE; dopachrome tautomerase	pigmentation; molecular function regulator; catalytic activity;
*RferTRXR1*	K20409	40,721– 42,259	DCE; dopachrome tautomerase	pigmentation; molecular function regulator; catalytic activity;
*RferLAC2*	k30924	176,411– 1,210,902	ALDH1L; formyltetrahydrofolate dehydrogenase	reproductive process; developmental process;

## Data Availability

The data supporting the findings of this study are provided in the manuscript and its Supplementary Materials. Additional information is available from the corresponding author upon reasonable request.

## Supplementary Materials

The following supporting information can be downloaded at https://www.mdpi.com/article/10.3390/biology15070555/s1, Figure S1: Morphological characteristics of male and female adult *Rhynchophorus ferrugineus*. (a) Female, dorsal view. (b) Female, lateral view. (c) Male, dorsal view. (d) Male, lateral view.
