# Supplementary material for: Exploring Speckle Change Genes of Rhynchophorus ferrugineus (Coleoptera: Curculionidae) Based on Genome-Wide Association Studies (GWASs)
Source: Biology (Basel). 2026 Mar 31;15(7):555. doi: 10.3390/biology15070555 (PMC13072381; doi:10.3390/biology15070555)
Supplement: Supplementary file 1 [file biology-15-00555-s001.zip › biology-4120609-supplementary.pdf]

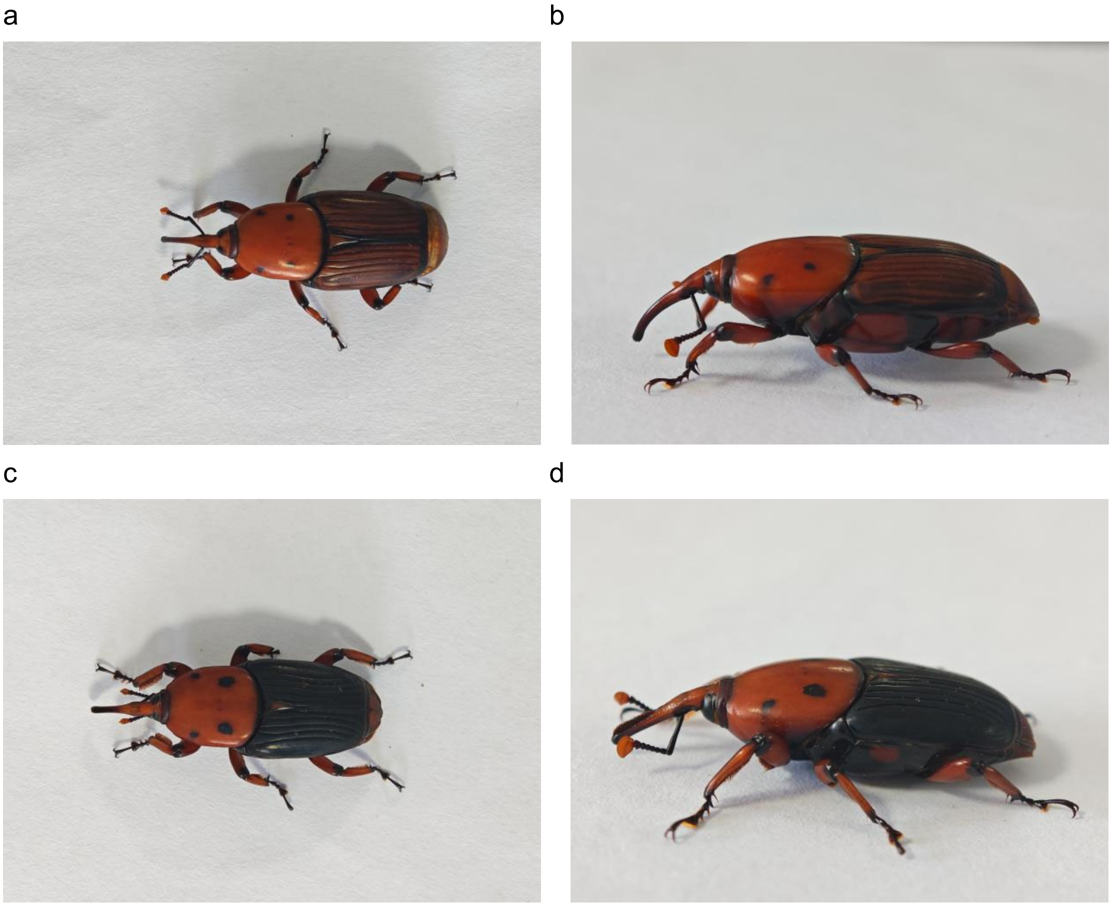

Supplementary Figure S1. Morphological characteristics of male and female adult *Rhynchophorus ferrugineus*. (a) Female, dorsal view. (b) Female,lateral view. (c) Male,dorsal view. (d)Male, lateral view.
